# What Do We Know about Classical and Non-Classical Progesterone Receptors in the Human Female Reproductive Tract? A Review

**DOI:** 10.3390/ijms222011278

**Published:** 2021-10-19

**Authors:** Yassmin Medina-Laver, Cristina Rodríguez-Varela, Stefania Salsano, Elena Labarta, Francisco Domínguez

**Affiliations:** 1 IVI Foundation—IIS La Fe, 46026 Valencia, Spain; yassmin.medina@ivirma.com (Y.M.-L.); cristina.rodriguez@ivirma.com (C.R.-V.); stefaniasalsano@gmail.com (S.S.); elena.labarta@ivirma.com (E.L.); 2 IVI RMA Valencia, 46015 Valencia, Spain

**Keywords:** progesterone, progesterone receptor, reproduction, female infertility, human

## Abstract

The progesterone hormone regulates the human menstrual cycle, pregnancy, and parturition by its action via the different progesterone receptors and signaling pathways in the female reproductive tract. Progesterone actions can be exerted through classical and non-classical receptors, or even a combination of both. The former are nuclear receptors whose activation leads to transcriptional activity regulation and thus in turn leads to slower but long-lasting responses. The latter are composed of progesterone receptors membrane components (PGRMC) and membrane progestin receptors (mPRs). These receptors rapidly activate the appropriate intracellular signal transduction pathways, and they can subsequently initiate specific cell responses or even modulate genomic cell responses. This review covers our current knowledge on the mechanisms of action and the relevance of classical and non-classical progesterone receptors in female reproductive tissues ranging from the ovary and uterus to the cervix, and it exposes their crucial role in female infertility.

## 1. Introduction

Progesterone (P4) is a steroid hormone synthesized by the placenta, ovaries, and adrenal glands. If we look at the etymology, the word progesterone comes from the Latin pro-, meaning “for,” and gest-, referring to pregnancy (as in “gestation”). Thus, it is commonly known as the “pregnancy hormone”. However, it also plays an important role in several non-reproductive tissues, such as the mammary gland to prepare breastfeeding, the cardiovascular system, the central nervous system, and bones [1].

In the female reproductive tract, P4 is involved in the regulation of the entire sequence of essential events that occur during the menstrual cycle and in pregnancy establishment and maintenance, such as ovulation, fertilization, implantation, embryonic development, and breast development, as well as parturition [2,3,4,5,6,7,8]. The high P4 levels present in the female reproductive tract during the periovulatory period also play a key role in sperm capacitation, hyperactivation, chemotaxis, and acrosome reaction [9], and they are crucial for optimal fertilization and subsequent embryo development.

Given its crucial role in female reproduction, impaired P4 signaling is usually associated with many gynecological complications, such as fibroids, abnormal menstrual bleeding, endometriosis, adenomyosis, breast and endometrial cancers, miscarriage, or preterm labor [2,3,10,11,12,13,14,15,16,17,18,19], not to mention that no successful pregnancy can be achieved with inadequate P4 action. For this reason, understanding how P4 exerts its action may contribute to developing more effective therapeutic approaches, improve the quality of life of those women with these disorders, and increase the chances of successful pregnancy and delivery.

The physiological effects of P4 are mediated by ligand binding to progesterone receptors (PGR) in target cells. These receptors are divided into classical and non-classical PGR [20]. Classical PGR, also known as nuclear PGR, have been found in the human ovary [21,22], uterus [23,24], fallopian tubes [24,25], placenta [26], testis [27], brain [28,29,30], pancreas [31], bone [32], mammary gland [12], and urinary tract [33]. Upon P4 binding, these transcription factors regulate the expression of P4 responding genes, which leads to a long-lasting, but slowly emerging, cellular response after several hours.

Unlike this delayed cellular response, P4 binding to non-classical PGR activates a wide variety of secondary messengers and signal transduction pathways by exerting rapid hormonal effects within seconds. These rapid responses are mediated by the activation of cell membrane receptors, cytoplasmic receptors, or classical receptor-independent intracellular signaling cascades [34]. These non-classical PGR have been found in several organs and tissues, including the female reproductive tract of different species [34,35,36,37,38,39,40]. Classical and non-classical signaling pathways have different downstream responses upon P4 binding, and they are responsible for the wide spectrum of P4′s different functions. Therefore, the cell response to P4 differs as to the type of signaling pathway which, in turn, is differently regulated in relation to tissue type and the moment of the menstrual cycle.

In this work, we review the different mechanisms of action described for classical and non-classical PGR, along with their main role in the different reproductive tissues and their implication in the final reproductive performance.

## 2. PGR Mechanisms of Action

### 2.1. Classical PGR

There are two main isoforms of classical PGR: PGR-A (94 kDa) and PGR-B (120 kDa). They are transcribed from the same gene by the utilization of two different promoters. In humans, the PGR gene consists of an eight-exon sequence located in chromosome 11 [41], and its transcription is usually estrogen-dependent. Upon the binding of 17β-estradiol or related estrogens to estrogen receptors (ER), the latter recognize several estrogen response elements (ERE) present in the promoter region of the PGR gene and induce its expression. Notwithstanding, several non-estrogen-dependent types of PGR expression regulation have also been described [42].

**Figure 1:**
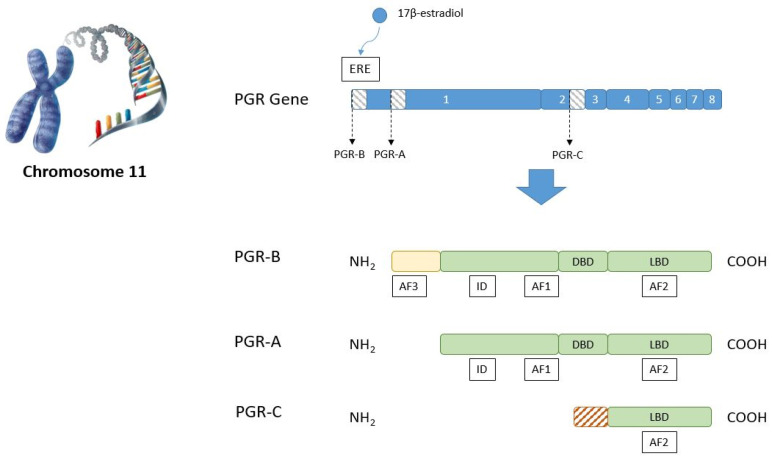
Representation of the classical PGR gene found in chromosome 11, and as of the protein domains of PGR isoforms A, B, and C. In response to estrogen binding to ERE, the PGR gene codifies for the distinct isoforms by the influence of different promoters. ERE = estrogen response elements. AF = activation function domain. ID = inhibitory domain. DBD = DNA-binding domain. LBD = ligand-binding domain. NH_2_ = amino terminal region. COOH = carboxyl terminal region.

The molecular structure of PGR consists of a DNA-binding domain (DBD) placed between an upstream N-terminal region containing an activation function (AF1) and an inhibitory domain (ID), and a downstream C-terminal region with a ligand-binding domain (LBD) which, in turn, contains another activation domain named AF2 (Figure 1). This is the same structure for isoforms PGR-A and PGR-B. However, PGR-B has an additional section located at the N-terminal end of the protein of approximately 164 extra amino acids in humans, which contains an additional activation domain called AF3 [43]. Several additional isoforms of PGR have been described, which result from the insertion of additional exons or are generated by alternative splicing. These variants may not encode a functional receptor and may, conversely, block or modulate the activity of the PGR-A and PGR-B isoforms [20]. Indeed, a third PGR-C isoform (60 kDa) has been described in the human placenta whose function is unclear, but it can form heterodimers with PGR-A and PGR-B by regulating their transcriptional activity [44].

**Figure 2:**
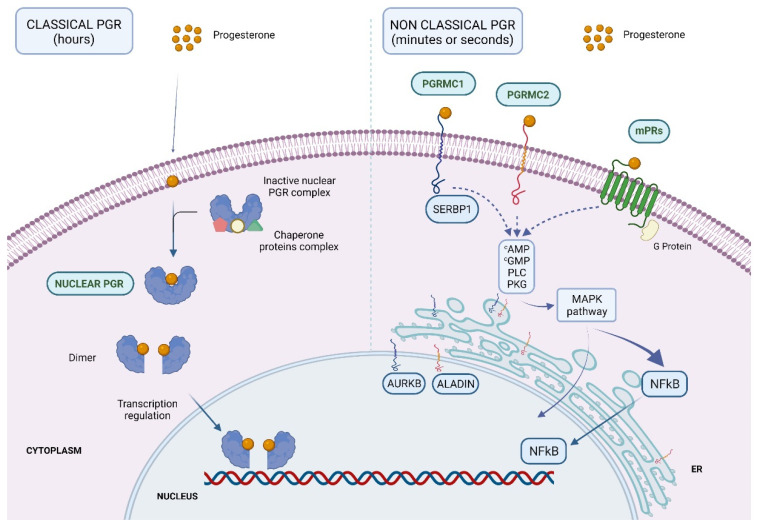
Graphic representation showing the subcellular localization and biological processes for classical (**left**) and non-classical (**right**) PGR in the cell and their interaction with proteins. ER = endoplasmic reticulum. Created with BioRender.com [47].

Unbound PGR are situated in the cytoplasm and linked with a complex of chaperone proteins. When P4 binds to the LBD, the receptor initiates a series of conformational changes and is released from the chaperone proteins to finally enter the nucleus. In the nucleus, PGR dimerizes and binds to the hormone response element sequence (HRE) in the promoter of the target gene. PGR-A and PGR-B can form homodimers (AA or BB) or heterodimers (AB), which gives rise to an extremely wide diversity of physiological responses. In addition, the binding of the PGR dimer to the HRE is followed by the recruitment of co-regulators, which can be co-activators or co-repressors, by regulating the subsequent PGR-mediated target gene expression (Figure 2).

Gene expression regulation by classical PGR is thought to be performed in an isoform-specific manner, although the specific mechanisms remain largely unknown. Several promoters have been described to be similarly regulated by both isoforms, more strongly or only by PGR-B, and more strongly or only by PGR-A [42]. Indeed, these differences in gene expression regulation lead to distinct physiological roles for these PGR isoforms as regards the target tissue, although the majority of normal human progesterone target tissues express similar amounts of PGR-A and PGR-B [45].

Regardless of this heterogeneous tissue and isoform-specific gene expression regulation, PGR isoforms also interact with one another by regulating their own activity. PGR-A inhibits PGR-B action by its inhibitory domain (ID), which decreases the effects of P4 on its target cells [46]. This is assumed to be the basis of a compensatory mechanism that is responsible for regulating P4 action throughout the ovarian cycle: higher P4 levels in luteal cells induce a higher PGR-A expression, which represses PGR-B transcription and, thus, cushions P4 effects. In contrast, lower P4 levels in these cells may suppress PGR-A expression, increase PGR-B expression, and subsequently enhance P4 action [20].

### 2.2. Non-Classical PGR

Non-classical P4 receptors are usually located on the cell surface and are structurally related to G protein-coupled receptors and single transmembrane receptors [35,48,49]. Consequently, they possess associated tyrosine kinase activity with subsequent MAP kinase (MAPK) pathway activation [50,51] (Figure 2).

**Figure 3:**
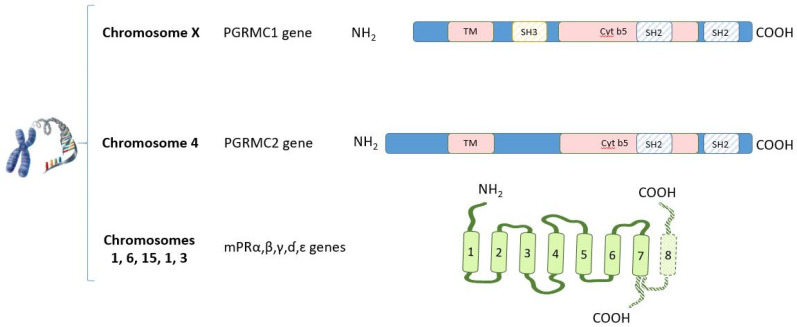
Schematic representation of the non-classical PGR genes and their localization in chromosomes. PGRMC1 and PGRMC2 comprise a single N-terminal transmembrane domain (TM) and a cytochrome (Cyt) b5 domain. The interaction sites for SH2 and SH3 domains revealed the presence of three binding sites for the Src homology domains. mPRα, β, and γ were proposed as being classical membrane G protein-coupled receptors with the typical 7 TM domain structure and the N-terminus facing the extracellular space. This assumption was challenged by placing an extended group of mPRs (α, β, γ, ɗ, and ɛ) with predicted 8 TM topology. NH_2_ = amino terminal region. COOH = carboxyl terminal region.

Non-classical P4 receptors can be divided into the membrane progestin receptors (mPRs) family and the progesterone receptor membrane component (PGRMC) family [36,52,53,54,55] (Figure 3).

Members of the mPRs family with putative P4-binding activity, also named Progestin and AdipoQ receptor (PAQR), belong to the G protein-coupled receptor superfamily and include membrane progesterone receptors α, β, γ, ɗ and ɛ) (also respectively called PAQR7, PAQR8, PAQR5, PAQR6, and PAQR9). Each gene has been cloned and partially characterized in mammals [56]. While these receptors have been shown to possess biological actions in vitro, others have questioned the valid function of these membrane receptors as PGR [57].

The second family of non-classical P4 receptors is the PGRMC family, which includes PGRMC1, PGRMC2, and the less studied neudecin, and also neuferricin. These non-classical PGR share a similar non covalent heme-binding domain related to cytochrome b5, which is a well-known functional interaction partner of microsomal cytochrome P450 (CYP) monooxygenase systems. The respective genes for the main studied non-classical PGR, PGRMC1, and PGRMC2 were originally cloned as heme-1 domain protein or HPR6.6 and Dg6, respectively [48,58,59]. Interestingly, the actions PGRMC and mPRs family members may be related because a physical interaction between PGRMC1 and mPRα has been demonstrated in cultured cells [60,61].

The physiological relevance of non-classical receptors is yet unclear, as their ability to bind P4 is relatively poor compared to classical PGR, and some studies suggest that they are not even activated by this hormone [62]. However, the non-classical actions of P4 have been shown to be associated with sexual behavior modulation in mice [63], meiotic maturation in Xenopus oocytes [64], the acrosomal reaction in human sperm [65], as well as the regulation of ion efflux in murine neurons [66], vascular myocytes [67], and rat epithelial cells [68].

The sections below discuss the main roles of these classical and non-classical PGR in reproductive tissues, particularly the ovary and uterus, along with their influence on reproduction. Table 1 summarizes the main human and animal studies that have assessed different PGR functions in reproductive tissues.

## 3. PGR in the Ovary and Oviduct

P4 action in the ovary and oviduct involves follicular development and recruitment, oocyte acquisition of competence, ovulation, and oviductal transport of cumulus–oocyte complexes (COC) and embryos [102]. This hormone also exerts feedback action on the hypothalamic–pituitary–gonadal axis by controlling the organism gonadal steroids’ secretion rate [28].

The ovarian function is influenced partly by alterations to the expression of many genes [103], such as PGR. High estrogen levels in the proliferative phase of the menstrual cycle lead to classical PGR expression upon the LH surge in granulosa cells of preovulatory follicles in the ovary [22], and also in the different cell types shaping the oviduct [24]. Upon ovulation, the arising corpus luteum starts P4 secretion, which interacts with the classical and non-classical PGR already present in these cell types. In humans, classical PGR expression is maintained during the active corpus luteum, but it ceases in the late corpus luteum [72]. Peluso et al. demonstrated that depleting PGRMC1 and PGRMC2 in luteal cells of mice alters the expression of growth factors, such as VEGF-a, Kit ligand and antimüllerian hormone (AMH) [104].

### 3.1. Role of PGR in the Hypothalamic–Pituitary–Gonadal Axis

PGR in the nervous system have been well-characterized in animal models [29]. P4 action extends beyond brain functions related to reproduction, such as neuroprotection, neuromodulation, myelination, neurogenesis, neuronal plasticity, mood, and early prenatal life neurodevelopment [29,30].

On the hypothalamic–pituitary–gonadal axis, P4 is responsible for the negative feedback that controls gonad hormonal secretion [28], and it exerts this action through classical and non-classical pathways.

In relation to the classical pathway, an increase in hypothalamic and pituitary tissue PGR-A and PGR-B levels has been proven after exogenous estradiol treatment in the rat model [69]. This finding suggests that once ovarian P4 production increases and high circulating P4 levels reach the nervous system, this hormone can decrease gonadotropin releasing hormone (GnRH) and luteinizing hormone (LH) release from the hypothalamus and pituitary, respectively, by means of PGR binding.

Conversely, a faster non-classical response to P4 is mediated by PGRMC1 but not mPRs expression in GnRH-secreting neurons in the hypothalamus. These receptors have been found to be implicated in P4 inhibition of GnRH neuronal activity through protein kinase G signaling [70]. Even though their direct action has not yet been proven, mPRs expression has been detected in the rat hypothalamus [71], which suggests a potential role of this receptor in P4 inhibition of GnRH release.

### 3.2. Role of PGR in Follicular Development and Recruitment

Classical PGR expression increases in granulosa cells from preovulatory follicles during the periovulatory period [22], which implies they play a crucial role in ovulation but not in follicular development and recruitment. Indeed, classical PGR knockout (PGRKO) mice have displayed normal ovarian follicle growth and development until the preovulatory stage [105].

Nevertheless, classical PGR have also been detected in a small proportion of human primordial and preantral follicles [72]. This implies a potential, albeit less studied, role of the P4 classical signaling pathway in follicular development and recruitment.

Regarding non-classical PGR, it is known that the conditional deletion of PGRMC1 interferes with mice antral follicle development, which evidences that PGRMC1 plays an important role in mammal antral follicle development [73,74]. Interestingly, P4 signaling through PGRMC1 inhibits primordial follicle formation in cultured neonatal mouse ovaries without lowering the total number of oocytes [76]. In vitro studies using human granulosa/luteal cells [61,74] or spontaneously immortalized granulosa cells (SIGCs) [73,106] demonstrate that PGRMC1 binds PGRMC2 and mPRα, which together partly regulate mitosis and apoptosis by suppressing the rate at which cells enter the cell cycle [75,77]. It has also been shown that PGRMC2 siRNA treatment does not reduce SIGCs’ capacity to bind P4 [73], unlike PGRMC1, whose depletion eliminates SIGCs’ ability to bind P4. Thus, PGRMC2′s capability to regulate P4 actions in SIGCs is dependent on PGRMC1, but the nature of this dependence remains unknown. Contrarily, PGRMC1 and PGRMC2 may also perform P4-independent actions because PGRMC1 siRNA in SIGCs increases several known genes that promote apoptosis when supplemental P4 is lacking [107,108,109].

The crucial role of PGR in follicular development and recruitment has been evidenced in women with decreased ovarian reserve and response to ovarian stimulation. On the one hand, low PGRMC1 expression has been found in women with premature ovarian insufficiency (POI) [80,81]. Moreover, decreased PGRMC2 expression has been observed in the granulosa cells of young women with diminished ovarian reserve [79]. The depletion of these receptors initiates inappropriate entry into the cell cycle, which often results in apoptosis [77] and, thus, probably reduces these women’s ovarian reserve. Later, Peluso et al. suggested that PGRMC1 and PGRMC2 regulate follicular cell cycle entry by precisely controlling the localization and, therefore, NFkB/p65 transcriptional activity. Such activity is promoted by apoptotic stimuli [78].

On the other hand, an altered PGRMC1 expression [110,111] in granulosa cells has been associated with a poor response to gonadotropin-induced follicle development in women undergoing in vitro fertilization.

### 3.3. Role of PGR in Ovulation

Following the LH surge, classical PGR action is crucial for successful oocyte release from the preovulatory follicle. However, experiments in PGR knockout mice have proven that classical PGR is required only for follicular rupture and not for the differentiation of granulosa cells into a corpus luteum [105].

Classical PGR perform their action during the follicular rupture process by regulating the transcription of several target genes, including the following: ADAMTS1, an extracellular matrix protease; endothelin-2 (EDN2), a vasoconstrictive peptide; epidermal growth factor-like (EGF-like) ligands, amphiregulin (AREG); and epiregulin (EREG) [102]. ADAMTS1 may play a role in the disruption of cellular interactions in the collagenous matrix or granulosa cells [112]. EDN2 is involved in smooth muscle contraction and follicular constriction by contributing to follicle rupture upon ovulation [113]. Finally, EGF-like ligands trigger cumulus expansion and subsequent meiotic resumption [114]. These target genes have been proposed after observing effects on ovulation in PGR knockout mice. However, the direct interaction of PGR with these genes and how it regulates their transcription have not yet been proven [102].

Mice knockout experiments have also helped to elucidate the specific roles of individual PGR isoforms in the ovulation process. Ovulation is completely absent in classical PGRKO mice, but it is only impaired in PGR-AKO mice, and it is not affected in PGR-BKO mice. This means that PGR-A expression is both necessary and sufficient for an efficient ovulatory response to P4. Histological analyses of PGR-AKO mice ovaries have revealed that their impaired ovulation may be due to PGR-B’s inability to mediate follicular rupture despite it being capable of regulating a subset of P4-responsive target genes [82].

On non-classical PGR, there is no evidence for its action during the ovulation process. Nevertheless, a recent study has demonstrated an abnormally lower metalloproteinase expression (including ADAMTS1, ADAMPTS8a, ADAMPTS9, MMP2, and MMP9) in double PGRMC1/2KO zebrafish, which led to a significantly lower ovulation rate [83].

The crucial role of PGR in follicular development, recruitment, and rupture has been evidenced in studies that have recruited women with polycystic ovarian syndrome (PCOS). This condition is characterized by a failure in dominant follicle selection and subsequent ovulatory disorders [115]. Therefore, disrupted PGR activity has been proposed in its pathophysiology. Indeed, low expression levels of ERs and classical PGR [116], as well as PGRMC1 [80], have been observed in granulosa cells from PCOS women after ovarian stimulation compared to normal cycling women.

### 3.4. Role of PGR in Oocyte Acquisition of Competence

The ovulation process is linked with meiotic arrest resumption in those oocytes enclosed in preovulatory follicles [117]. Given the important role of classical and non-classical PGR in follicular development, it is feasible to assume that these receptors may also play a key role in the acquisition of oocyte competence. However, meiotic resumption is a process that depends on cumulus cells’ expansion and, unlike granulosa cells, classical PGR expression in cumulus cells is very low [118].

In the animal model, some PGRKO mice experiments are in favor of [119] and against [105] an important role for classical PGR in oocyte maturation and developmental competence. In humans, no relation has been found between classical PGR expression in cumulus cells from IVF patients and oocyte fertilization or cleavage rate. However, the same study found an association between low classical PGR expression and good embryo quality [84].

In contrast, many studies have proved the involvement of the non-classical pathway in mammalian and teleost oocyte maturation. Terzaghi et al. demonstrated that PGRMC1 participates in late bovine mitosis and oocyte meiosis events by interacting with AURKB (an essential protein to properly complete oocyte meiosis [86,120]), which is consistent with PGRMC1 localization in the mid-zone and mid-body of mitotic and meiotic spindles, as other groups have previously observed [85,86,121,122]. In addition, PGRMC2 participates in murine meiotic spindle assembly by interacting with ALADIN [87], which is a nucleoporin involved in both meiotic and mitotic divisions [123,124].

On the involvement of mPRs in oocyte maturation, several authors have demonstrated a relevant role of mPRα in oocyte maturation regulation and embryo development in bovine and teleosts [37,125,126,127]. They suggest that mPRs may also influence P4 intracellular signaling by its interaction with classical and other non-classical PGR, which is important for oocyte developmental competence and, consequently, for successful pregnancy.

### 3.5. Role of PGR in Oviductal Transport of COCs and Embryos

Upon ovulation, released oocytes travel through the oviduct to the uterine cavity. Oviductal transport of COCs and embryos is highly regulated by steroid levels during the menstrual cycle. In the follicular phase, higher estradiol levels accelerate this transport, while higher P4 levels in the luteal phase decelerate it [128]. Hormonal action controls this transport by acting at three different levels: ciliary beating; muscular contraction; and fluid volume and composition [102]. In this line, P4 decreases ciliary beat frequency [128], promotes muscular relaxation [129], and avoids fluid accumulation in the luteal phase in the oviduct [130]. However, the specific PGR role in these P4-mediated actions at the oviductal level in humans remains unknown. It has been suggested that the co-expression of classical and non-classical PGR in human oviductal cells provides the possibility of a cooperative relation in mediating cilia function [102].

In the animal model, classical and non-classical PGR expression have been proven in the bovine, canine, and cattle oviduct. Interestingly, Hazano et al. showed that the bovine expression levels of classical PGR and PGRMC2 were higher in the isthmus epithelium than in the ampulla, whereas PGRMC1 expression displayed the opposite pattern [88]. In contrast, other bovine studies have reported similar [90] and higher PGRMC2 expression levels in the ampulla [89] than in the isthmus. Finally, differential PGR expression has been proven in the canine oviduct [91].

Taken together, these findings suggest that P4 may exert its actions by different receptors regarding the oviduct part, despite controversy about the predominant signaling pathway in each section. Further studies are necessary to elucidate PGR function in human fallopian tube transport.

## 4. PGR in the Uterus

P4 action induces several changes in uterine cells, which lead to endometrial receptivity acquisition and take part in the embryo implantation process [131]. This means that when the developing embryo reaches the uterus, it finds an already prepared optimal uterine environment to implant. The crucial role of this hormone is proven by the fact that estrogen/P4 therapy alone suffices to support viable pregnancy after donor embryo transfers in postmenopausal women [132].

In the human uterus, P4 action has been described following both classical [82] and non-classical [40] signaling pathways. The expression of classical and non-classical PGR in the uterus significantly oscillates throughout the menstrual cycle by regulating all the critical events that finally establish a receptive endometrial microenvironment and correct pregnancy [95,133,134].

Longer estrogen exposure in the follicular phase induces the classical PGR expression in uterine cells. In the subsequent luteal phase, P4 exposure inhibits ER expression, which leads to estrogenic drive that, in turn, augments P4 responsiveness. Although the role of non-classical PGR in the uterus is not well-known, its expression has been documented in human, rhesus monkey, mouse, zebrafish, and bovine uterus [11,94,95,109,135,136,137,138,139]. Nevertheless, classical and non-classical PGR isoforms behave differently depending on the uterine cell type and the moment of the menstrual cycle.

P4 acts in the uterus at different levels because classical and non-classical PGR levels are detectable in epithelial and stromal/decidual cells in the endometrium [140], smooth muscle cells in the myometrium [141], and stromal fibroblasts in the cervix [142]. The next sections discuss P4 actions at these different uterine levels and in relation to whether pregnancy is achieved or not.

### 4.1. Role of PGR in the Endometrium

The endometrium is formed mainly of epithelial and stromal cells. In the luteal phase, P4 action in these cell types inhibits their proliferation, which is previously enhanced by estrogen action in the preovulatory phase. In contrast, P4 promotes morphological and functional changes by establishing a glandular secretory epithelium and a vascular stroma as part of a process called decidualization [131], which occurs in endometrial stromal cells (EnSCs) [143,144]. This transformation makes the endometrium receptive and ready for the embryo to be implanted.

If pregnancy is not achieved, a drop in P4 levels is due to luteolysis reverse decidualization, which induces a process involving endometrial inflammation, cell death, and extracellular matrix degradation that leads to menstruation [145]. If pregnancy takes place, maintained P4 levels promote the decidualization process completion in the endometrium [146].

Classical PGR expression differs as regards endometrial cell type, and this difference is thought to be mediated by cell type-specific variations in PGR-A and PGR-B expressions and functions [131]. In the endometrial epithelium, both PGR-A and PGR-B are expressed prior to embryo implantation. At this point, the inhibitory effect of PGR-A on PGR-B expression controls the action of the latter receptor in promoting hyperplasia and inflammation in this tissue [147]. However, during implantation, PGR-A levels drop and PGR-B levels remain constant to control glandular secretion. Conversely, in EnSCs, PGR-A acts as the predominant isoform throughout the luteal phase because it participates in the decidualization process [140].

Mice PGRKO experiments have shown that the expression of PGR-A, but not of PGR-B, suffices for successful implantation and pregnancy [82]. However, the overexpression of this isoform has also been associated with the enlargement of the uterus and endometrium hyperplasia [148]. Hence, the PGR-A/PGR-B expression ratio needs to be accurately regulated for a normal endometrial epithelium and stroma response to P4.

Indeed, a recent study with a group of infertile patients showed those with unexplained infertility had the lowest endometrial epithelial expression levels of both PGR-A and PGR-B [92]. Moreover, the recurrent implantation failure (RIF) phenomenon has been associated with not only lower classical PGR expression in the endometrium [93] but also with the expression of other classical PGR polymorphisms [149]. In particular, the so-called PROGINS polymorphism leads to amino acid substitution in exon 4. This allele produces a classical PGR with increased transcriptional activity and stability, and its presence has been related to a reduction in several endometrial receptivity markers [150].

Non-classical PGR have been observed to also be expressed in the endometrium of different mammals, such as human, mouse, rhesus monkey, and bovine, as we previously mentioned. In most of them, these receptors also oscillate during the menstrual cycle to prepare the endometrium for embryo implantation.

In humans, PGRMCs, mPRα, mPRγ, and mPRε transcripts have been demonstrated to significantly vary according to the menstrual cycle phase [11,94,95]. In particular, PGRMC1, mPRγ, and mPRε transcripts are up-regulated in the proliferative phase and progressively decrease in the secretory phase, whereas mPRα and PGRMC2 mRNA are significantly overexpressed in the secretory phase and concur with the postovulatory rise in P4 [11,94,95].

mPRβ are relatively more abundant in the human endometrium than mPRα but do not change significantly during the menstrual cycle [94]. Nevertheless, a reduction in the endometrial mPRβ gene expression on days 10–14 of the human menstrual cycle has been seen in patients with a history of recurrent spontaneous abortion [151].

PGRMC1 and PGRMC2 show inverse expression patterns during the menstrual cycle, which is probably due to a gene expression regulation phenomenon of the former over the latter. PGRMC1 is involved in cell proliferation and thus is responsible for endometrium development in the first half of the menstrual cycle [108]. In fact, its ablation results in reduced fertility in female mice [96]. In the secretory phase, PGRMC1 stromal overexpression inhibits decidualization and creates an improper embryo implantation environment [95]. In contrast, PGRMC1 down-regulation in this phase may also impair uterine receptivity and blastocyst implantation [152]. Therefore, balanced PGRMC1 levels are necessary for proper endometrial receptivity. Garrido-Gómez et al. demonstrated that PGRMC1 behaves differently in human receptive endometrium vs. a non-receptive endometrium [97]. Salsano et al. detected PGRMC1 movement from the cytoplasm to the nucleus when the decidualization process occurred in humans [95]. This contrasts with the expression of the classical PGR, which is known to be absent in the endometrial stroma upon embryo implantation [153]. This interesting nuclear localization suggests a possible function for PGRMC1 in directly regulating the expression of a specific set of distinct genes from the target genes of classic classical PGR during decidualization. Alternatively, PGRMC1 stroma nuclear localization may lead to participation in cell cycle regulation when proliferative stromal cells are in transition to terminally differentiated decidual cells.

PGRMC2 inhibits cell proliferation and thus its presence in the secretory phase. The specific role of PGRMC2 in the decidualization process has not yet been elucidated even though its deficiency causes premature uterine senescence in PGRMC2 knockout (PGRMC2KO) mice, unlike normal senescence that occurs in the physiological decidualization process [154,155] and leads to postimplantation failure [96,156]. A similar scenario has been observed in PGRMC1 knockout (PGRMC1KO) and PGRMC2KO zebrafish [138].

Endometrial PGR dysregulation causes several reproductive diseases such as endometriosis. With this condition, endometriotic tissue undergoes growth and morphology changes during the menstrual cycle in parallel to the eutopic endometrium [157]. Therefore, its proliferation is induced by estrogen exposure in the proliferative phase and is inhibited by P4 in the secretory phase. P4 resistance may cause this abnormal endometrial proliferation [158]. One possible explanation for this P4 resistance is abnormal classical PGR signaling in the ectopic endometrium. In this context, low classical PGR expression in the ectopic endometrium of patients with endometriosis, compared to eutopic tissue, has been reported in the literature [159], even though studies are equivocal [160]. Bearing this in mind, it has been hypothesized that this abnormal signaling arises in the eutopic endometrium to confer it a predisposition to form ectopic implants. A recent study has shown a reduced PGR-B, but similar PGR-A, expression in the eutopic endometrium of women with endometriosis versus a control group without endometriosis [161].

Additionally, reduced PGRMC2, mPRα, mPRβ, and mPRγ expression has been observed in endometrial hyperplasia and endometriosis [11,162,163]. This finding confirms the role of PGRMC2 up-regulation in the secretory phase and how P4 acts through these receptors to inhibit cell proliferation. Nonetheless, it contradicts the role of mPRγ in promoting proliferation in the proliferative phase during the human menstrual cycle [94].

### 4.2. Role of PGR in the Myometrium

The myometrium is composed of myometrial smooth muscle cells and is the tissue responsible for uterine contractions. In the proliferative phase, higher estrogen levels stimulate peristaltic waves of myometrial contractions, and the direction of these waves goes predominantly from the cervix to the fundus and favors sperm transport to the fertilization zone [164]. Conversely in the secretory phase, P4 relaxatory action decreases the frequency and intensity of these waves, and it favors oocyte transport to the uterine cavity and its approach to the implantation site [4,165].

If implantation has not occurred in the late luteal phase, the fall in P4 levels increases myometrial contractility and leads to menstruation. Yet if pregnancy is achieved, the maintained high P4 levels exert their relaxatory action on myometrial cells and avoid uterine contractions and parturition [118]. As with menstruation, delivery is induced by functional P4 withdrawal in human myometrial cells [141,166,167].

Myometrial cells express both PGR-A and PGR-B throughout the menstrual cycle and pregnancy, and P4′s action via these receptors regulates myometrium contractile activity. This is evidenced by the fact that administering the classical PGR antagonist, RU486, increases uterine contractions in the secretory phase [98] and also induces labor and delivery if administered during pregnancy [99].

PGR-B seems to possess progestational actions in these cells, and PGR-A mainly regulates PGR-B expression levels [131]. Indeed, the parturition process is thought to be mediated by a switch in the PGR-A:PGR-B ratio in favor of PGR-A (with PGR-C an up-regulation), which may inhibit PGR-B progestational actions by increasing myometrial contractility and excitability, and leading to labor [168].

The P4 non-classical pathways’ faster action is believed to directly affect myometrial contraction by either modulating intracellular signal transduction pathways [4,54] or inhibiting calcium influx [169]. Several studies about the myometrial contractility of pregnant women have demonstrated the presence of mPRα, mPRβ, PGRMC1, and PGRMC2 in human myometrial cells [57,94,100,170].

Changes in these receptors could potentially contribute to functional P4 withdrawal in the human myometrium during labor. PGRMC2 and PGRMC1 expression levels are significantly lower in in-labor women than into non-in-labor women [170]. In contrast, Wu et al. found no significant differences in PGRMC1 mRNA expression, contrarily to protein expression, between term and preterm myometria. Despite no statistically significant differences in mRNA expression having been found, they demonstrate a downward trend for PGRMC1 mRNA from term non-labor to the term labor myometrium. In addition, the pretreatment of myometrial strips with a PGRMC1 antibody suppresses P4-induced relaxation [100].

Regarding mPRs, Fernandes et al. observed an expression pattern of these receptors in human parturition, similarly to that described for PGRMCs in the previous paragraph. They found a significant reduction in myometrial mPRα expression during preterm and term labor, while mPRß expression reduced only during term labor [94,171]. These findings contrast with those reported by Karteris et al., who described the up-regulation of mPRα, albeit not mPRβ, in human myometrium during labor [57].

A recent study has suggested crosstalk between classical and non-classical PGR during gestation and labor. The authors hypothesized that mPRs induce PGR-B transactivation, which is essential for maintaining myometrial quiescence and cervical closure during gestation [57,172]. At the end of pregnancy, mPRα and mPRß may activate the p38 MAPK pathway to induce the phosphorylation of Myosin Light Chain and to down-regulate SRC2 expression (a PR-B coactivator) by triggering labor and delivery [57].

Accordingly, classical and non-classical PGRs are relevant for human myometrium contractility, which is an important event for obtaining proper embryo attachment and parturition.

### 4.3. Role of PGR in the Cervix

In the proliferative phase, cervical cells produce a thin watery mucus in response to estrogen, which allows the passage of sperm to the uterus. In the secretory phase, P4 induces the production of a viscous cervical mucus, which forms a plug that restricts the passage of sperm from the vagina. If pregnancy is achieved, cervical closure in response to P4, as well as increased collagen production and rigidity, become even more pronounced [131]. P4 action is also thought to play a major role in preventing preterm birth, especially in women with premature cervical shortening [173].

These effects are largely mediated by classical PGR, which are expressed in the stromal fibroblasts and basal squamous epithelial cells of the human cervix [101]. P4 action through the classical pathway in these cells promotes cervical closure and collagen production by regulating the expression of the genes related to collagen synthesis and breakdown and by also antagonizing estrogen-induced collagenase expression [131].

Despite their unknown contribution to physiological P4 action in the human cervix, non-classical PGR seem to play a key role in premalignant and malignant diseases of the female cervix. For instance, lower PGRMC2 levels have been detected in nodal metastasis of uterine endocervical adenocarcinomas, which suggests a potential role of this receptor as a tumor suppressor [174], while mPRs have been detected in cervical cancer cell lines HeLa [54] and C4-I [162].

## 5. Conclusions

Progesterone has long since been considered the hormone of pregnancy. Technological advances and the use of animal models have extensively enhanced our understanding of how P4 regulates the human menstrual cycle, pregnancy, and parturition by its action via PGR. These receptors exert pleiotropic effects by regulating many types of proteins, including ligands, receptors, chaperones, signaling proteins, and transcription factors. Understanding these processes is vital to make progress in not only reproductive physiology, but also in pathology, such as endometriosis and endometrial cancer.

This review has shown that P4 action can be mediated through classical and non-classical receptors, or even a combination of both. In the ovary, PGR are involved in oocyte release during ovulation, and also in the transport of gametes and embryos throughout the oviduct. It also suggests an important role of these receptors in oocyte acquisition of competence. In the uterus, PGR are expressed in the endometrium, myometrium, and cervix. P4 action by these receptors promotes endometrial receptivity, embryo implantation, and pregnancy maintenance, which it does mainly by either promoting endometrium decidualization, myometrium relaxation, and cervical closure or maintaining a physiological condition.

The dysregulation of this set of PGR leads to many reproductive diseases, such as PCOS, POI, endometriosis, implantation failure, etc. Hence, identifying other novel PGR targets involved in the regulation of many P4 functions, most of which are yet to be identified, as well as their interactions, would be extremely beneficial for the diagnosis and treatment of female infertility.

## Figures and Tables

**Table 1:** List of the main human and animal studies to have assessed PGR functions in reproductive tissues: ovary and oviduct, and uterus. C = classical. NC = non classical.

Reproductive Tissue	Role	Study	Model	Type of PGR	Main Conclusions
**OVARY and** **OVIDUCT**	Gonadal neurological regulation	[69]	Rat	C	Increased PGR-A and PGR-B levels in hypothalamic and pituitary tissue after exogenous estradiol treatment
		[70]	Mouse	NC	PGRMC1 is implicated in the P4 inhibition of GnRH neuronal activity
		[71]	Rat	NC	mPRs expression has been detected in the hypothalamus
	Follicular development and recruitment	[22]	Human	C	Increased expression in granulosa cells from preovulatory follicles during the periovulatory period, which suggests their role in ovulation, but not in follicular development and recruitment
		[72]	Human	C	Detected in a small proportion of human primordial and preantral follicles
		[73,74]	Mouse	NC	PGRMC1 and PGRMC2 interfere with antral follicle development
		[75]	A. croaker	NC	mPRα mediates the antiapoptotic actions of progestins in ovarian follicle cells
		[76]	Mouse	NC	PGRMC1 mediates P4-induced suppression of oocyte development and primordial folliculogenesis
		[77]	Mouse	NC	PGRMC1 and PGRMC2 interact to suppress entry into the cell cycle in spontaneously immortalized granulosa cells
		[78]	Rat	NC	PGRMC1 and PGRMC2 regulate granulosa cell mitosis and survival through an NFkB-dependent mechanism
		[79,80,81]	Human	NC	Lower PGRMC1 and PGRMC2 levels in women with premature ovarian insufficiency and decreased ovarian reserve
	Ovulation	[82]	Mouse	C	Ovulation was completely absent in classical PGRKO mice, but it was only impaired in PGR-AKO mice and unaffected in PGR-BKO mice
		[83]	Zebrafish	NC	Lower metalloproteinase expression and ovulation rate in double PGRMC1/2KO zebrafish
	Oocyte acquisition of competence	[84]	Human	C	No relation between the classical PGR expression in cumulus cells of IVF patients and oocyte fertilization or cleavage rate. Association between reduced classical PGR expression and good embryo quality
		[85]	Rat, Human	NC	PGRMC1 regulates spindle microtubule stability during rat and human ovarian cell mitosis
		[86]	Bovine	NC	PGRMC1 participates in late mitosis and oocyte meiosis events and interacts with AURKB
		[87]	Mouse	NC	PGRMC2 participates in meiotic spindle assembly with ALADIN
		[37]	Bovine	NC	Role of mPRα in oocyte maturation and embryo development regulation
	Oviduct transport	[88,89,90,91]	Bovine Canine	C and NC	Classical PGR, PGRMC1, and PGRMC2 expression in different parts along the oviduct
**UTERUS**	Endometrium	[82]	Mouse	C	PGR-A expression, but not PGR-B, is sufficient for successful implantation and pregnancy
		[92]	Human	C	Infertile patients with unexplained infertility with the lowest levels of endometrial epithelial expression of both PGR-A and PGR-B
		[93]	Human	C	Association of recurrent implantation failure with decreased classical PGR expression in the endometrium
		[11,94,95]	Human	C and NC	PGRMC1, mPRγ, and mPRε transcripts are up-regulated in the proliferative phase and progressively decrease in the secretory phase, whereas mPRα and PGRMC2 mRNA are significantly overexpressed in the secretory phase. mPRα do not significantly change during the menstrual cycle.
		[96]	Mouse	NC	Conditional ablation of PGRMC1 results in female subfertility
		[97]	Human	NC	PGRMC1 behaves differently in a receptive vs. a non-receptive endometrium
	Myometrium	[98,99]	Human	C	P4 action via these receptors regulates myometrium contractile activity
		[57,94,100]	Human	NC	Presence of mPRα, mPRβ, PGRMC1, and PGRMC2 in myometrial cells
	Cervix	[101]	Human	C	Expressed in stromal fibroblasts and basal squamous epithelial cells of the cervix
